# LEHP-DETR: A model with backbone improved and hybrid encoding innovated for flax capsule detection

**DOI:** 10.1016/j.isci.2024.111558

**Published:** 2024-12-09

**Authors:** Changshun Wang, Junying Han, Chengzhong Liu, Jianping Zhang, Yanni Qi

**Affiliations:** 1 College of Information Science and Technology, Gansu Agricultural University, Lanzhou 730000, China; 2 Crop Research Institute, Gansu Academy of Agricultural Sciences, Lanzhou 730000, China

**Keywords:** Bioinformatics, Plant biology, Agricultural science

## Abstract

Flax, as a functional crop with rich essential fatty acids and nutrients, is important in nutrition and industrial applications. However, the current process of flax seed detection relies mainly on manual operation, which is not only inefficient but also prone to error. The development of computer vision and deep learning techniques offers a new way to solve this problem. In this study, based on RT-DETR, we introduced the RepNCSPELAN4 module, ADown module, Context Aggregation module, and TFE module, and designed the HWD-ADown module, HiLo-AIFI module, and DSSFF module, and proposed an improved model, called LEHP-DETR. Experimental results show that LEHP-DETR achieves significant performance improvement on the flax dataset and comprehensively outperforms the comparison model. Compared to the base model, LEHP-DETR reduces the number of parameters by 67.3%, the model size by 66.3%, and the FLOPs by 37.6%. the average detection accuracy mAP50 and mAP50:95 increased by 2.6% and 3.5%, respectively.

## Highlights

• Propose a superior model with improved backbone and hybrid encoding innovation
• Give a new idea for easily and efficiently detecting Flax Capsules by deep learning
• Lightweight model for precise flax capsule detection in agriculture and industry
• The HWD-ADown, Hilo-Attention, and DSSFF modules were developed to improve the model

## Graphical abstract

**Figure:**
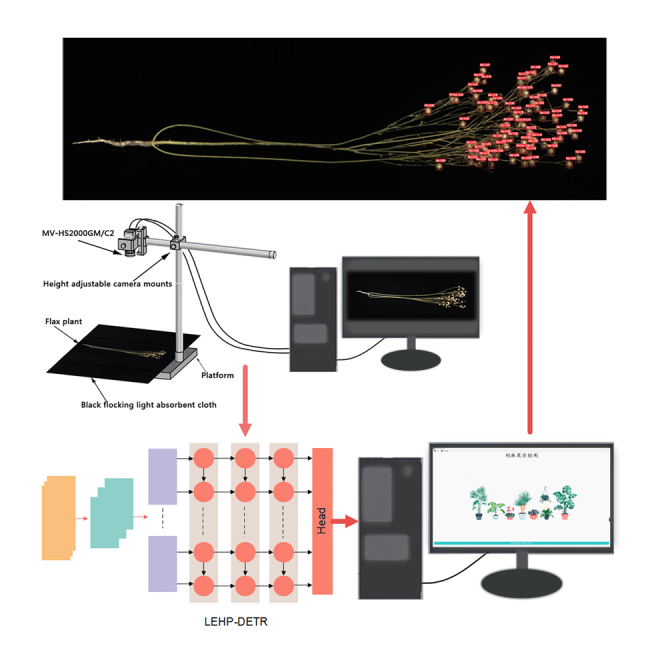

## Teaser

Bioinformatics; Computer science; Artificial intelligence; Agricultural science

## Introduction

Flax, because it is rich in essential fatty acids and nutrients, has become more and more popular with consumers and is recognized by international nutritional and physiological scientists as one of the important functional crops; At the same time, but also because of the toughness of its fiber, it is an important industrial and textile fabric. The innovation of germplasm resources and the cultivation of new special varieties are the fundamental ways out for the healthy and sustainable development of the flax industry, and rapid and accurate seed detection is one of the most important bases for the development of the flax industry. At present, flax seed detection relies mainly on manual labor but with low efficiency, a high error rate, and a certain degree of subjectivity. With the accelerated process of urbanization, labor costs continue to increase, which seriously limits the development of the flax industry; how to use computer technology to achieve convenient, efficient, and accurate seed detection has obtained the focus of global flax breeding experts.

In recent years, the development of computer vision and deep learning techniques have provided new ways of solving this problem, in particular, object detection,^1^^,^^2^ which has become an important area of research in the field of agriculture.^3^^,^^4^ Object detection techniques provide the basis for visual analysis by locating and identifying objects in an image. Xiang et al.^5^ proposed an object detection model called YOLO POD for soybean pod counting, which enables fast and accurate counting of soybean pods. Although this model is guaranteed in terms of accuracy, parameters and the number of floating point operations (FLOPs) are still large. Li et al.^6^ conducted a comparative study of detecting agricultural greenhouses using high-resolution satellite imagery by three models, Faster R-CNN,^7^ YOLOv3,^8^ and SSD.^9^ The results of this study show that YOLOv3 has significant advantages in both accuracy and computational efficiency. However, this study has some obvious limitations, i.e., only comparing these three models’ performances, but no comparative study with other mainstream models, YOLOv4 and YOLOv5. Li et al.^10^ proposed an improved version of the YOLOv3-based model, named L-YOLO, for lemon fruit detection. In the lemon test set, L-YOLO showed excellent performance with an average accuracy of 96.28% at a detection speed of 90 FPS, indicating that the detection model has a significant advantage in terms of efficiency. However, as lemons are relatively large, L-YOLO may not be as effective at detecting small objects as it is at detecting large objects. Chen et al.^11^ proposed an improved model based on the YOLO-v4^12^ model, called optimal YOLO-v4, specifically for the detection of bayberry trees in UAV images. The model showed excellent performance with a detection accuracy of 97.78% and a recall of 98.16%. However, the model also suffers from some loss of accuracy. When the background elements exhibit characteristics akin to the object features of the bayberry tree, such as analogous colors, comparable shapes, and equivalent textures, the model may false detect or omit them, resulting in a loss of accuracy. Hu et al.^13^ proposed an Object Relation Module (ORM) for modeling the relationship between two objects in an image. This module consists of modeling the appearance correlation and geometric correlation of the objects. In this way, the ORM is able to incorporate this relationship information into the detection head of the two-stage detector and in post-processing, which not only improves the accuracy of object recognition but also enables an end-to-end processing flow, making the use of the model easier and more efficient. Lim et al.^14^ proposed a small object detection method that combines contextual information and attention mechanisms to significantly improve the accuracy and performance of small object detection. The core of the method is to improve small object detection by linking multiscale features and using additional features from different layers as contextual information. In addition, the method introduces an object detection technique with an attention mechanism that effectively focuses on the object in the image and integrates contextual information from the object layer. In complex and challenging environments, the limited low-resolution, high-pixel information of small objects makes the use of contextual information particularly critical. This approach not only significantly improves the detection accuracy of small objects but also increases the robustness and adaptability of the model, enabling it to better cope with diverse detection scenarios. Guo et al.^15^ proposed a scale-adaptive small object detection method in complex agricultural environments using honeybees as a research object. The method aims to overcome the influence of the complex and variable background environment as well as the difficulty of extracting small object features to achieve small object detection. The specific method includes splitting the original image into smaller subgraphs to increase the object scale, assigning the labeled objects to the split subgraphs to form a new dataset, and then re-training and generating a new object recognition model using a migration learning approach. The recall and precision of the proposed scale-adaptive algorithm are generally higher than those of the SSD model, up to 3.8% and 2.6%, respectively. However, the algorithm requires multiple forward inferences for a single image, resulting in the timeliness of the model is low. Sun et al.^16^ used the YOLO-V5^17^ model to detect moldy areas in rice and estimate the area covered by mold. The results showed that the developed YOLO-v5 model exhibits high accuracy and recall. This method, grounded in computer vision and YOLO convolutional neural networks, offers a promising solution for the rapid identification of mold in rice during distribution and trade.

In summary, the detection of small objects in the field of agriculture still faces a number of challenges, including the difficulties of feature selection, the loss of feature information, and the problem of false detection and missing detection due to different responses to features at various scales.

These problems are particularly significant in flax capsule detection because the number of capsules hanging on the flax plant is much more than other plants, and the number of flax capsules of a plant can even reach more than 150. But all the capsules are different from each other in shape and size, because the ovary chamber of the flax capsule may vary from 6 to more, resulting in the size, the shape, and the outline of flax capsule being irregular. Also, the branch numbers of each flax plant are much more and the branches randomly grow in different positions and directions, which leads to the capsules being easily covered by branches, leaves, and other capsule. In addition, all capsules are not ripe at the same time; some capsules have fallen off only with the withering receptacles, some capsules have not cracked, but some capsules have slightly cracked, and some capsules even have completely cracked. All these above characteristic details about the flax plant make it more difficult to detect flax capsules in breeding examination. However, research in this field is still in its early stages; especially, the combination of deep learning for small object detection of flax capsules is rarely reported. Therefore, there is an urgent need for relevant research to fill this gap.

The performance of object detection models based on CNN architecture,^18^ such as the YOLO series,^8^^,^^12^^,^^17^^,^^19^^,^^20^ although they perform well in most real-time detection applications, is greatly limited for flax capsule recognition. Transformer^21^^,^^22^^,^^23^^,^^24^ architecture RT-DETR^25^ can overcome the above difficulties in the detection of flax capsules, and effectively avoid the false detection and missed detection problems caused by the YOLO series NMS algorithm,^26^ providing new possibilities for plant breeding in the flax field.

In this study, an improved model LEHP-DETR, based on the transformer architecture RT-DETR, is proposed to address the challenge of detecting small objects in flax capsules. The main contributions of this research are reflected in the following aspects.

(1) In this study, the Haar wavelet downsampling-ADown (HWD-ADown) module was innovatively designed to effectively retain critical edge information while reducing image resolution. This design takes into account the low contrast and susceptibility to occlusion of small objects, such as flax capsules in the image, with the aim of improving object detection accuracy. In addition, the RepNCSPELAN4 module^20^ and the ADown module^20^ were also introduced in this study to replace the original backbone network. By combining these three modules, a lightweight model and efficient inference speed are achieved while effectively improving the feature representation and reducing the feature map resolution, further reducing the computational complexity and improving the performance of the model in small object detection tasks.
(2) In this study, the Context Aggregation module is introduced to more efficiently integrate features from different modules and to improve the ability of the model to exploit the contextual information of the object in the image.^27^ The introduction of this module takes into account the importance of global information, which helps to understand the overall structure and context of the image, as well as the capture of local information, which helps to identify details and specific features in the image. With this module, the model is able to locate and identify small objects more accurately, especially in complex and changing agricultural production environments.
(3) In this study, two key modules are designed and implemented: the HiLo Attention-based Intrascale Feature Interaction Module (HiLo-AIFI) and the DySample Scale Sequence Feature Fusion Module (DSSFF). The HiLo-AIFI module improves the processing speed and efficiency of the model by optimizing the allocation of the attention head and the computational method, which reduces the computational burden while maintaining high performance. At the same time, the module is able to capture different frequency features in the image more comprehensively, which gives the model better generalization ability to different types of images and scenes. The DSSFF module is able to better fuse features of different scales through the scale sequence feature fusion module and, therefore, has higher performance when dealing with multiscale object detection tasks. This design helps the model to identify and locate objects more accurately in complex scenes and dynamically adjusts the size of the feature map according to the needs of different tasks, thus improving the performance of the model. In addition, the Triple Feature Encoding module (TFE)^28^ is introduced in this study. It improves the detection of dense and small objects by splicing three different feature sizes in the spatial dimension to capture detailed information about flax capsules.
(4) In this study, the P2 detection head is introduced to address the problem of small objects occupying fewer pixels in the image and being easily obscured by other larger objects or the background. The P2 detection head is located at a shallower layer of the network and is able to capture feature information at a lower level. These low-level feature maps typically contain more detail and edge information, and the design of the P2 detection head helps to improve the visibility of these small objects, allowing the model to locate and identify them more accurately.^12^

## Results

**Figure 1:**
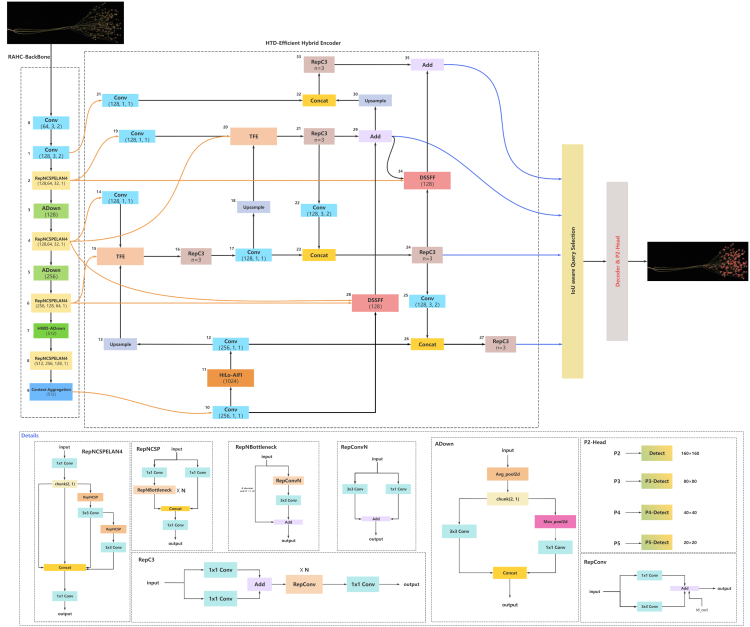
Structure of the LEHP-DETR network

**Figure 2:**
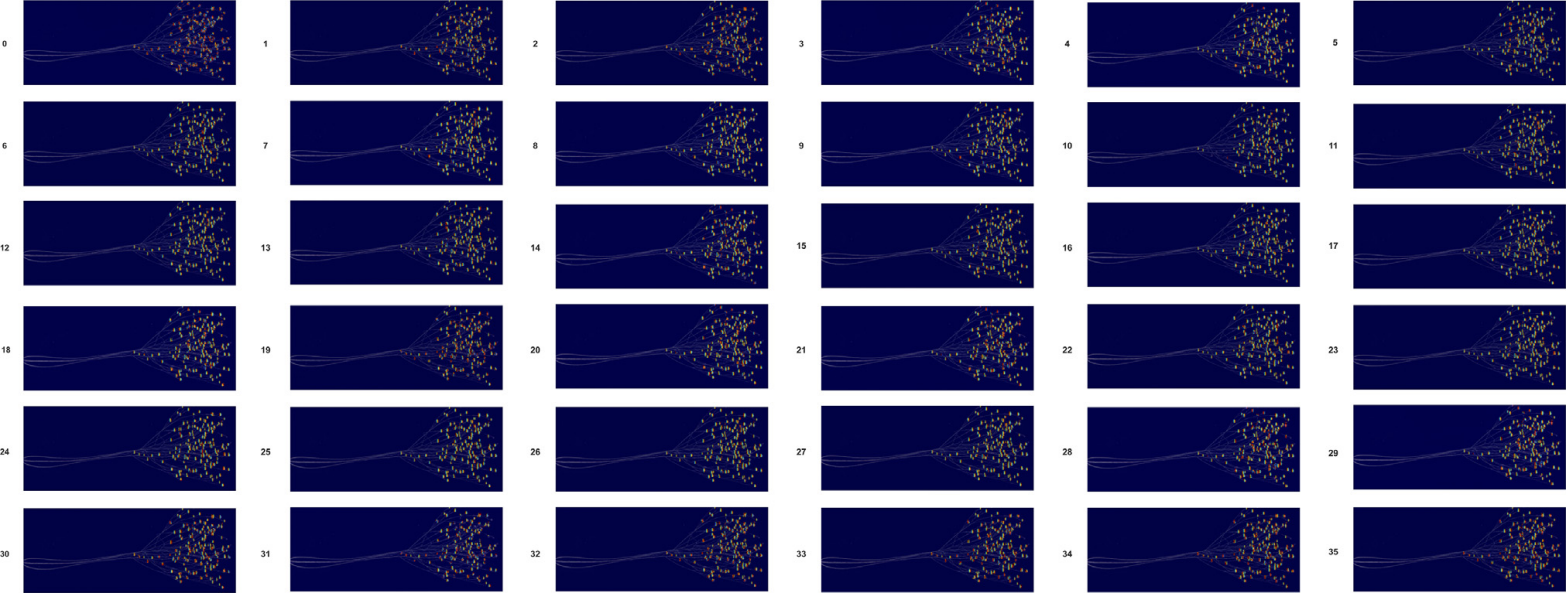
Heat maps for each layer of the LEHP-DETR model 0–35 Corresponding to Figure 1 respectively.

In this study, a novel object detection model, LEHP-DETR, is proposed, which is a further improvement of the R18 model based on RT-DETR. The structure of the model is shown in Figure 1, which consists of three main parts: the RAHC-BackBone, the HTD-Efficient Hybrid Encoder, and the P2-Head. The RAHC-BackBone improves the traditional backbone network by introducing the RepNCSPELAN4 module, the ADown module, and the designed HWD-ADown module, thus improving the feature expression capability and reducing the computational complexity. This modification not only achieves a lightweight model but also improves the inference speed, especially in the small object detection task. HTD-Efficient Hybrid Encoder designs the HiLo-AIFI module and DSSFF module and further introduces the TFE module to achieve the overall improvement of the model performance. The HiLo-AIFI module improves the generalization ability of the model by distinguishing between high-frequency and low-frequency information to better capture the local details and global structure of the image. The DSSFF module enriches the detailed information by integrating feature maps of different scales and enhances the model’s multi-scale feature extraction capability. The TFE module accurately captures the detailed information of small objects by combining features of different sizes in the spatial dimension. In addition, the Context Aggregation module is introduced between the RAHC-BackBone and the HTD-Efficient Hybrid Encoder, which aims to strengthen the model’s understanding and use of contextual information, as well as to optimize the feature supply process to ensure that the high-level feature maps can better retain the detail information in the low-level feature maps. In the P2-head component, an innovative P2-detection head is integrated, capitalizing on the abundant detail and edge information present in the low-level feature maps. This enhancement markedly amplifies the detection efficacy for small objects. In order to analyze the role of each module in the model, a LayerCAM^29^ heatmap visualization technique was used to reveal the output of each layer of the model and its impact on the detection task of flax capsule. As shown in Figure 2, we can visually see the contribution of different layers to the detection of flax capsule through the heatmap, so as to provide a basis for model optimization and performance improvement. The LEHP-DETR model achieves a balance between real-time performance and detection accuracy, and through its innovative structural design, it demonstrates a significant performance advantage in the task of detecting small objects in flax capsules.

### Comparative experiment

In order to comprehensively validate the efficiency and accuracy of LEHP-DETR in the task of object detection, this study compared it with current mainstream models. The experiments were carried out on the FLAX dataset and the DOTA dataset^30^ to validate its efficiency and accuracy in different scenarios. The models involved in the comparison include the base model RT-DETR-R18, YOLOv5m, YOLOv8m, and YOLOv9c. To ensure that the comparison remains fair and validated, the experimental platforms and datasets of all five models are kept consistent. In this study, YOLOv5m, YOLOv8m, and YOLOv9c used the model and code version provided by the Ultralytics team, choosing AdamW^31^ as the optimizer, which can further improve the stability and performance of model training. default learning rate (lr) and momentum using adaptive parameters by the Ultralytics team, epoch of 300, and ensuring better convergence and higher accuracy of the model, will disable mosaic augmentation of the final epoch, from the default of 10–120. RT-DETR-R18 and LEHP-DETR use the parameters described under model training parameters in the Experimental Studies section. In order to compare models fairly, none of the comparative models above used pre-training weights.

**Figure 3:**
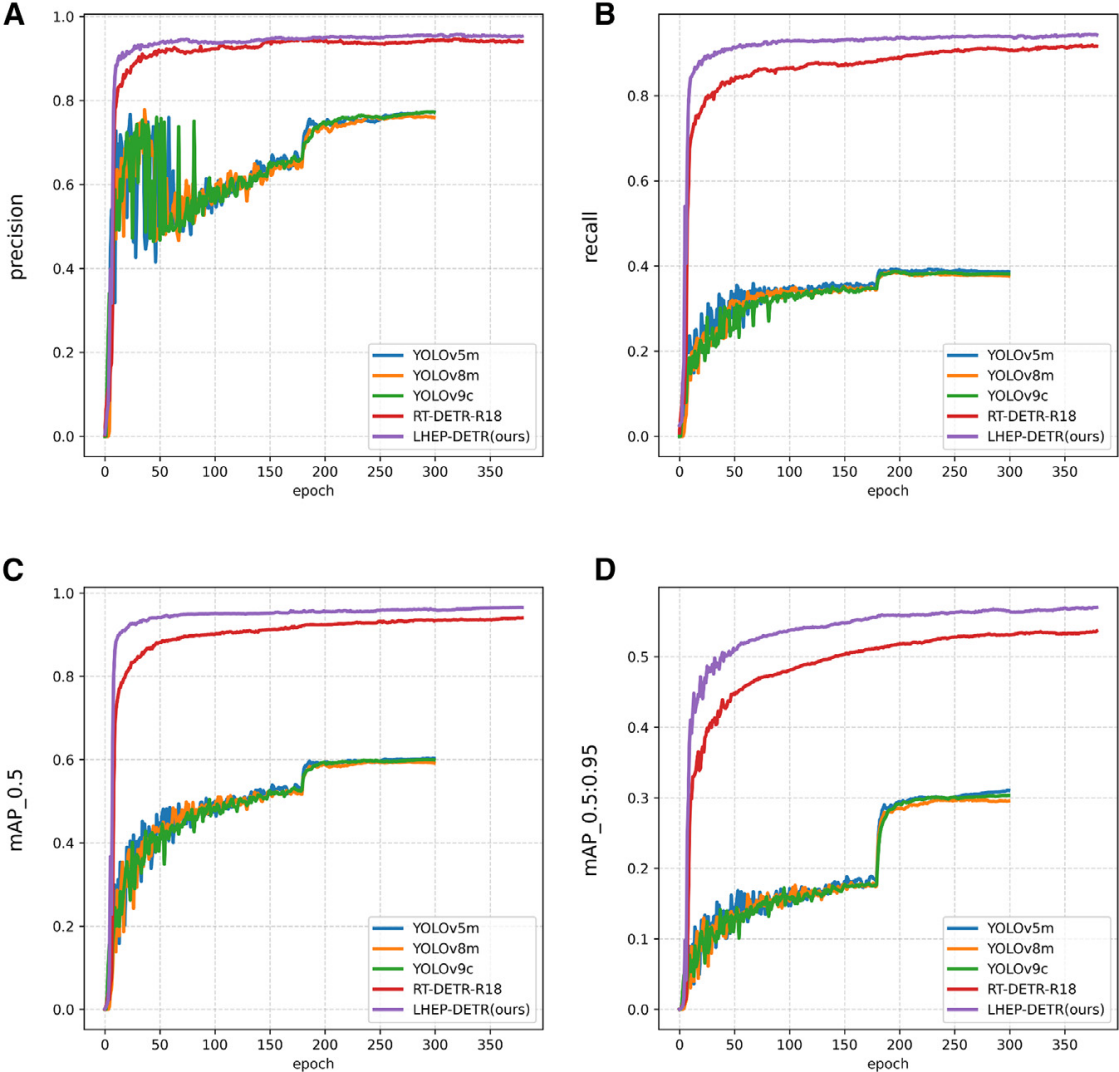
Comparison of model-experiment results on FLAX dataset (A) Precision curve, (B) Recall curve, (C) mAP 0.5 curve, (D) mAP 0.5:0.95 curve.

**Figure 4:**
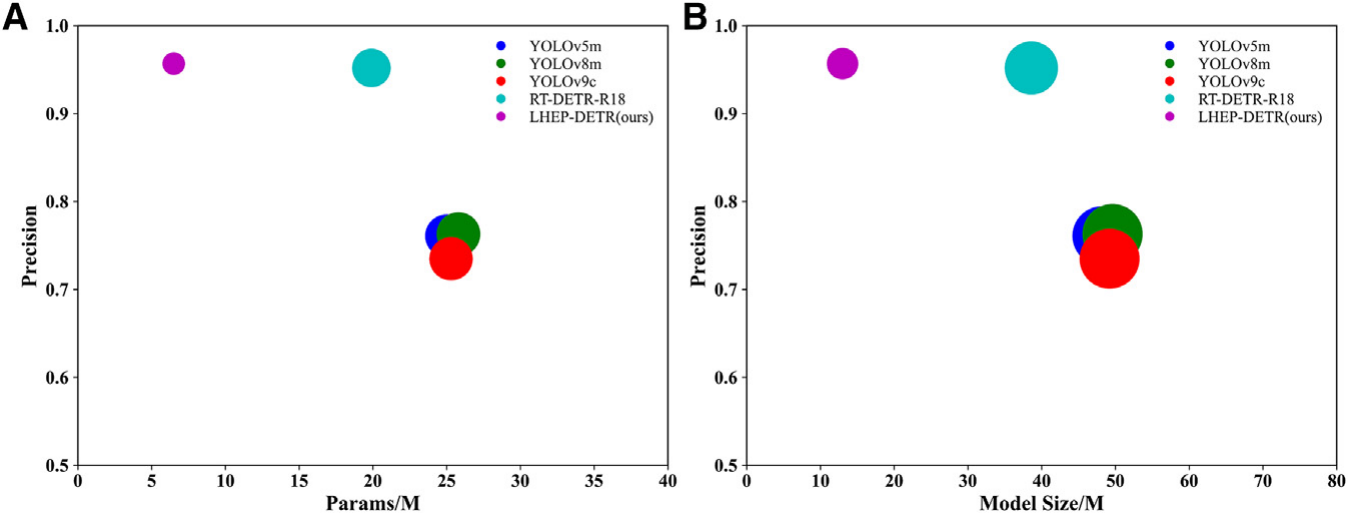
Accuracy versus model size and parameters on FLAX dataset (A) Model Accuracy and number of parameters of the model on FLAX dataset. the Size of the circles represents the size of the attribute value. (B) Model Accuracy and model size on FLAX dataset. The Size of the circles represents the size of the attribute value.

On the flax dataset As shown in Table 1, Figures 3 and 4, this study compares the specific performances of different models under uniform evaluation metrics. The new model LHEP-DETR proposed in this study achieves a significant improvement in all the indices with 96.9% accuracy, 95.2% recall, 98.0% mAP50, and 61.3% mAP50:95 while minimizing the model size, a number of parameters, and FLOPs computation, which comprehensively outperforms the other compared models. Although the YOLO series model has a certain advantage in speed, the model in this study still achieves an FPS of 260.3, which shows good real-time performance and meets the real-time requirements.

**Table 1:** Comparison experiments of different models on FLAX dataset

Model	Model Size (M)	Params (M)	FLOPs (G)	Precision (%)	Recall (%)	mAP50 (%)	mAP50:95 (%)	FPS bs = 16
YOLOv5m	48.2	25.0	64.0	77.4	36.0	59.0	30.9	**465.4**
YOLOv8m	49.6	25.8	78.7	77.0	35.9	58.7	30.2	398.2
YOLOv9c	49.2	25.3	102.3	78.0	36.2	59.4	30.7	272.4
RT-DETR-R18	38.6	19.9	56.9	95.9	92.0	95.4	57.8	391.6
LEHP-DETR (Ours)	**13.0**	**6.5**	**35.5**	**96.9**	**95.2**	**98.0**	**61.3**	260.3

Significant values are in bold.

**Figure 5:**
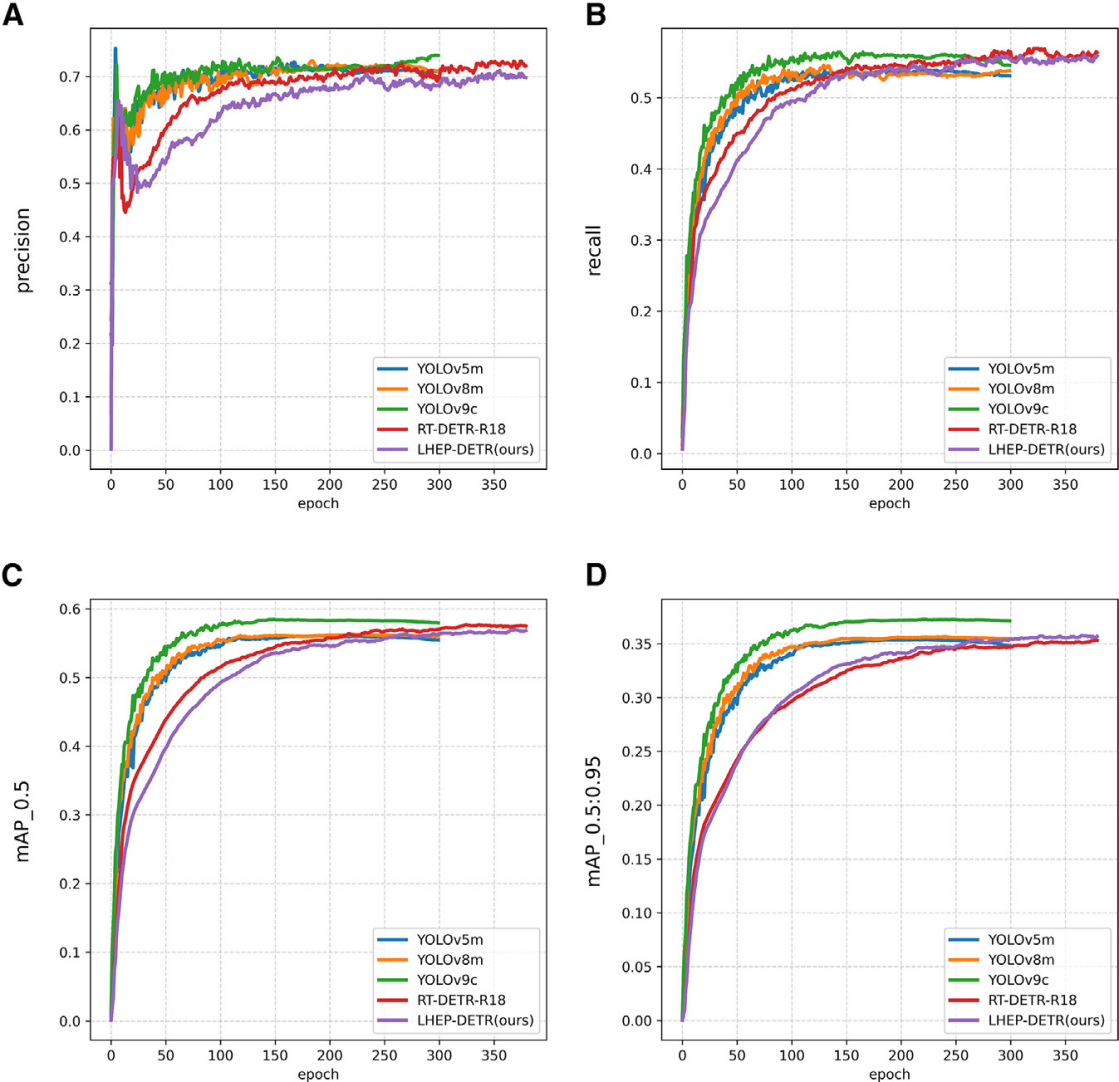
Comparison of model-experiment results on DOTA dataset (A) Precision curve, (B) Recall curve, (C) mAP 0.5 curve, (D) mAP 0.5:0.95 curve.

**Figure 6:**
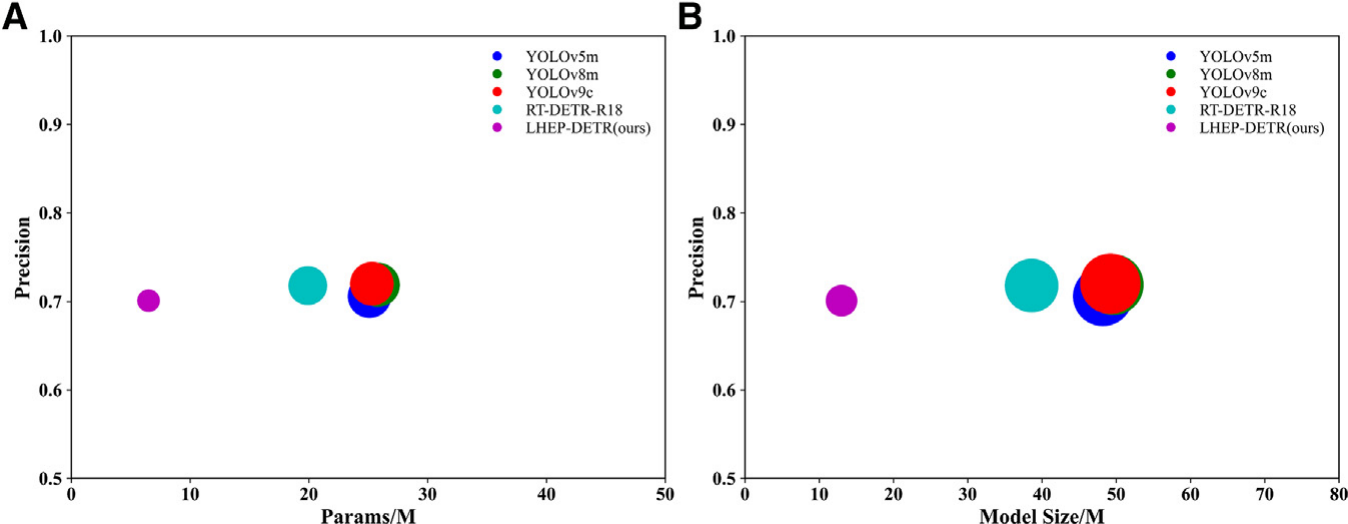
Accuracy versus model size and parameters on DOTA dataset (A) Model Accuracy and number of parameters of the model on DOTA  dataset. the Size of the circles represents the size of the attribute value. (B) Model Accuracy and model size on DOTA dataset. The Size of the circles represents the size of the attribute value.

On the DOTA dataset as shown in Table 2, Figures 5 and 6, the model size of LEHP-DETR is much lower than all other models compared, which is especially important for resource-constrained devices. The number of parameters is much lower than the other models, and although LEHP-DETR is comparable to the other models in terms of precision and recall, its FLOPs are 35.6G, which is significantly lower than the other models. This suggests that LEHP-DETR is computationally more efficient and is able to run with fewer computational resources. LEHP-DETR significantly reduces the model size and computational complexity while maintaining similar precision, recall, mAP50, and mAP50:95 as the other models. Although the FPS of LEHP-DETR is lower than the comparison model at a batch size of 16, it still achieves 260.8 FPS, which meets the demand of real-time detection.

**Table 2:** Comparison experiments of different models on DOTA dataset

Model	Model Size (M)	Params (M)	FLOPs (G)	Precision (%)	Recall (%)	mAP50 (%)	mAP50:95 (%)	FPS bs = 16
YOLOv5m	48.2	25.1	64.0	70.6	53.9	56.0	35.4	466.9
YOLOv8m	49.6	25.8	78.7	71.9	53.1	56.2	35.7	398.0
YOLOv9c	49.2	25.3	102.4	72.0	55.6	58.3	37.3	272.0
RT-DETR-R18	38.7	19.9	57.0	71.8	56.5	57.5	35.3	394.6
LEHP-DETR (Ours)	**13.0**	**6.5**	**35.6**	70.1	55.5	56.9	35.8	260.8

Significant values are in bold.

Through the comparison experiments, this study demonstrates that the new model proposed in this study achieves excellent object detection performance while maintaining lightweight and efficiency.

### Cross-validation test

When performing statistical significance tests to verify whether the performance improvement is due to random factors, this study uses a 10-fold cross-validation method to enhance the reliability of the results. By dividing the FLAX dataset into 10 subsets and using 7 subsets for training, 2 subsets for testing, and 1 subset for validation in each iteration. This study ensures the robustness of the evaluation, thus excluding chance results due to sample selection or specific subsets. This approach helps to reduce bias and increases our confidence in the significance of model performance improvement.

In Table 3, the results of the 10-fold cross-validation clearly show that the LEHP-DETR model performs relatively consistently across the different data subsets and outperforms all comparison models overall. The model shows high stability in terms of precision and FPS, but there are some fluctuations in the recall and mAP50:95 metrics. This fluctuation may be due to the unevenness of the dataset or the sensitivity of the model to some specific samples. Nevertheless, LEHP-DETR significantly reduces the size and computational complexity of the model while maintaining high performance, making its deployment in real applications more flexible and efficient.

**Table 3:** Ten-fold cross-validation of LEHP-DETR on FLAX dataset

Performance	1st	2st	3st	4st	5st	6st	7st	8st	9st	10st	Min	Max	Avg	Std
Model Size (M)	13.0	13.0	13.0	13.0	13.0	13.0	13.0	13.0	13.0	13.0	–	–	–	0
Params (M)	6.5	6.5	6.5	6.5	6.5	6.5	6.5	6.5	6.5	6.5	–	–	–	0
FLOPs (G)	35.5	35.5	35.5	35.5	35.5	35.5	35.5	35.5	35.5	35.5	–	–	–	0
Precision (%)	97.0	96.5	97.1	97.2	97.3	96.8	97.2	96.4	97.2	97.0	96.4	97.3	96.97	0.309
Recall (%)	95.8	95.3	95.5	95.3	95.5	96.1	95.1	94.2	95.8	95.9	94.2	96.1	95.45	0.538
mAP50 (%)	98.2	98.0	98.1	98.1	98.2	98.2	98.1	97.7	98.2	98.3	97.7	98.3	98.11	0.166
mAP50:95 (%)	61.8	60.9	62.6	62.5	61.9	62.1	62.4	61.3	60.8	63.3	60.8	63.3	61.96	0.792
FPS bs = 16	259.4	258.8	259.0	259.1	259.1	259.6	259.4	259.4	259.6	260.0	258.8	260	259.34	0.350

Cross-validated data are denoted by - when it is the same. avg stands for average. std stands for sample standard deviation. min stands for minimum. max represents the maximum value.

In summary, the LEHP-DETR model demonstrates good stability and generalization ability in 10-fold cross-validation, while maintaining high resource efficiency and real-time performance. These characteristics make LEHP-DETR a valuable model in practical applications, suitable for deployment in resource-constrained environments, and capable of meeting the demands of real-time detection.

### Practical comparison

**Figure 7:**
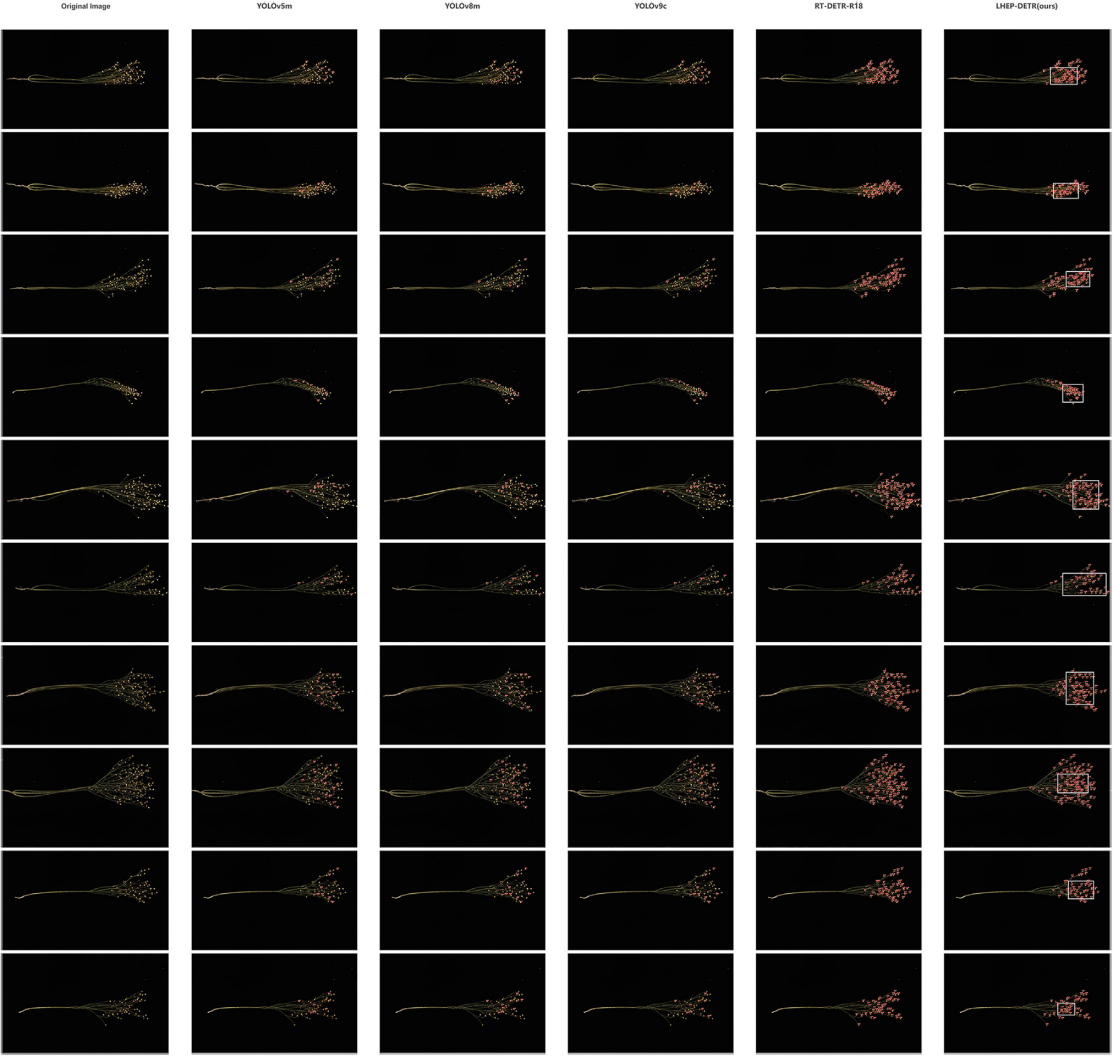
Comparison of YOLOv5m, YOLOv8m, YOLOv9c, RT-DETR-R18 and LHEP-DETR Practical Applications on FLAX dataset

**Figure 8:**
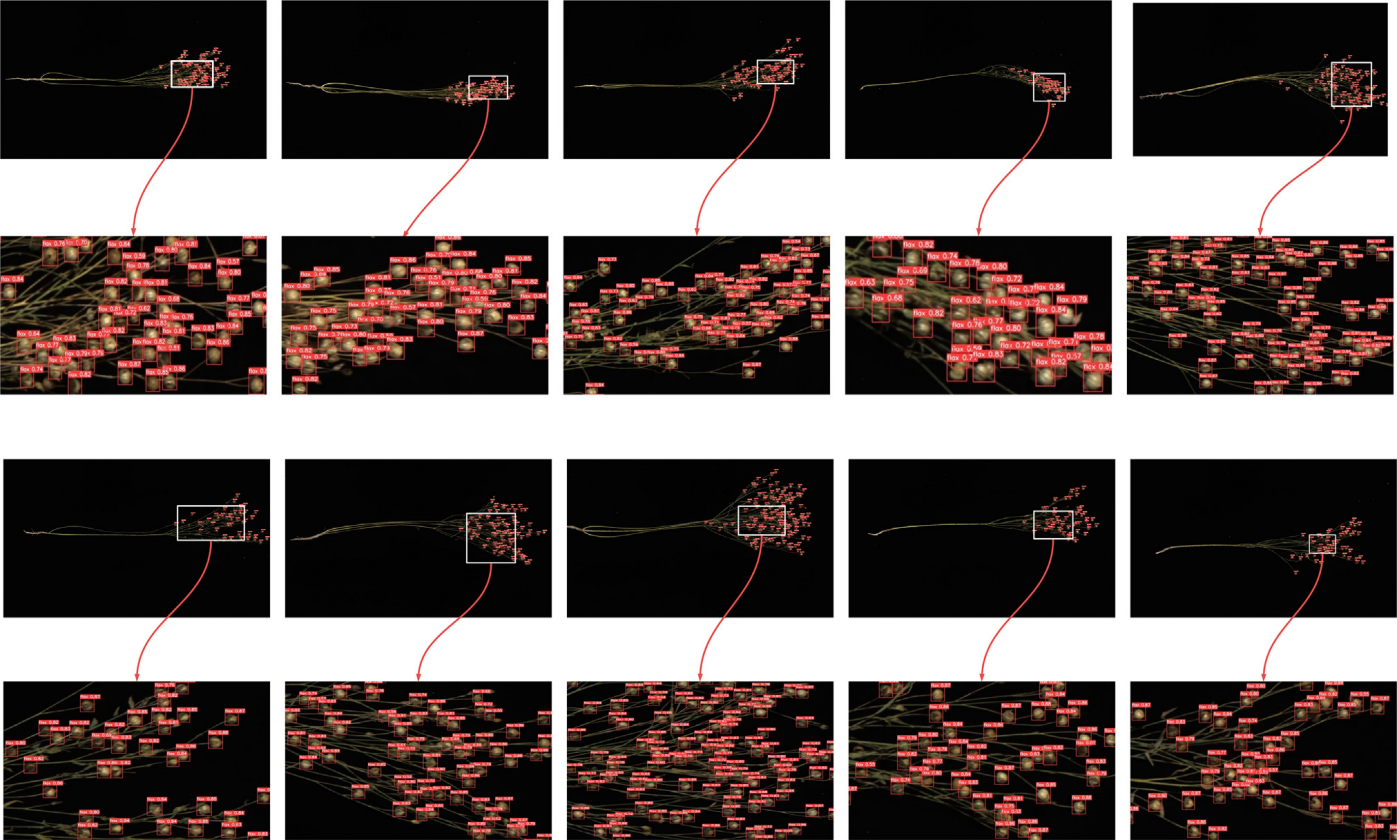
Local zoomed detail in practical application of the LHEP-DETR model on FLAX dataset

In order to evaluate the performance of the model in practical applications, ten external images not included in the original dataset were carefully selected in this study to simulate real-world application scenarios. Through the visual display in Figure 7, this study can clearly observe that the YOLOv5m, YOLOv8m, and YOLOv9c models have significant missed detections in object detection tasks, and the overall detection performance is poor, making it difficult to meet basic object detection requirements. In contrast, the new model LEHP-DETR proposed in this study shows excellent performance in the object detection task, achieving almost complete detection of all objects with extremely rare leakage and false detection. During the object detection process, this study used white framing for the detection of details of interest in the images and local zooming to clearly demonstrate the model's detection capability. As shown in Figure 8, this visualization method helps to analyze the performance of the model in detail. This practical comparison verifies that the new model proposed in this study is reliable and stable for real-world application scenarios. Therefore, LEHP-DETR provides a new, effective solution for easy, efficient, and accurate object detection tasks in real applications.

### Model efficiency verification on multiple hardware platforms

In order to further validate the lightweight and efficient nature of the LEHP-DETR model, this study conducted FPS measurements on three typical hardware platforms to provide a more comprehensive support of efficiency in real-world applications. Specifically, the study conducted performance tests on laptop, desktop, and workstation using central processing unit (CPU) and graphics processing unit (GPU), respectively. The results are summarized in Table 4.

**Table 4:** LEHP-DETR FPS measurements on multiple hardware platforms batch size (bs) is 1

Hardware Platforms	Configurations	FPSbs = 1	
		CPU	GPU
Laptop	CPU:Intel Core i9-12900HX 12th Gen GPU:NVIDIA GeForce RTX 4060 Laptop with 8 GB	4.5	61.4
Desktop	CPU:Intel Xeon W-2123 GPU:NVIDIA GeForce GTX 1080 with 8 GB	1.5	24.8
Workstation	CPU:Intel Core i7- 13700 13th Gen GPU:NVIDIA GeForce RTX 4090 with 24 GB	5.4	92.4

### Ablation experiment

In order to evaluate the impact of different modules in the RAHC-BackBone model on model performance, this study conducted ablation experiments. Table 5 shows the performance indicators of the model under different configurations. On the basis of the RT-DETR-R18 model, this study gradually integrated RA (joint action of RepNCSPELAN4 and ADown module), HA (HWD-ADown module), and CA (ContextAggregation module). The results of the ablation experiment show that compared to the benchmark model, the A1 configuration reduces model size, parameters, and FLOPs while improving frame rate (FPS), proving that the RA module enhances model performance while maintaining computational efficiency. B1 configuration is based on A1; although the model size, the parameters, and the number of FLOPs have slightly increased, there have been improvements in recall, mAP50, mAP50:95, and FPS indicators, demonstrating the further promoting effect of the HA module on model performance. The C1 configuration achieved further improvements in precision, mAP50:95, and FPS metrics while maintaining the size and number of parameters of the B1 model, confirming the enhancing effect of the CA module on model performance. Taken together, this study concludes that the RA, HA, and CA modules for RAHC-BackBone achieve a smaller model size and also improve inference speed.

**Table 5:** Effect of individual modules in the RAHC-BackBone model on model performance

Model	RA	HA	CA	Model Size (M)	Params (M)	FLOPs (G)	Precision (%)	Recall (%)	mAP50 (%)	mAP50:95 (%)	FPS bs = 16
RT-DETR-R18	×	×	×	38.6	19.9	56.9	95.9	92.0	95.4	57.8	391.6
A1	✓	×	×	**20.8**	**10.4**	**33.4**	**95.9**	91.7	94.8	55.6	**445.1**
B1	✓	✓	×	22.4	11.3	34.1	95.6	**92.4**	95.7	56.3	431.1
C1	✓	✓	✓	22.5	11.3	34.1	95.8	92.0	95.6	**56.6**	441.4

RA denotes the RepNCSPELAN4 module in conjunction with the ADown module, HA denotes the HWD-ADown module, and CA denotes the ContextAggregation module. On FLAX dataset. Significant values are in bold.

In order to deeply explore the specific effects of HiLo-AIFI and TDS-CCFM modules on the HTD-Efficient Hybrid Encoder model, this study carried out a series of ablation experiments. The results of these experiments are summarized in Table 6 for a comprehensive assessment and analysis of the impact of each module. The TDS-CCFM module is composed of DSSFF and TFE modules. In the A2 configuration, this study integrated the HiLo-AIFI feature on the base model, and the results showed that although FLOPs were slightly increased, the model size and precision remained the same, and the recall, mAP50:95, and FPS were significantly improved to 92.4%, 58.0% and 398.3, respectively. It indicates that HiLo-AIFI has a positive impact on model performance. B2 configuration adds the TDS-CCFM feature on the basis of A2, which further improves the precision to 96.1%, and recall, mAP50, mAP50:95 indicators are essentially identical. Although FPS decreased from 398.3 to 305.6, it still meets the real-time requirements. These results show that the introduction of TDS-CCFM features increases the computational complexity of the model, but it also significantly improves the performance of the model.

**Table 6:** Effect of individual modules in the HTD-Efficient Hybrid Encoder model on model performance

Model	HiLo-AIFI	TDS-CCFM	Model Size (M)	Params (M)	FLOPs (G)	Precision (%)	Recall (%)	mAP50 (%)	mAP50:95 (%)	FPS bs = 16
RT-DETR-R18	×	×	38.6	19.9	56.9	95.9	92.0	95.4	57.8	391.6
A2	✓	×	**38.6**	**19.8**	57.1	96.0	**92.4**	**95.8**	**58.0**	**398.3**
B2	✓	✓	39.2	20.2	61.5	**96.1**	92.2	95.6	57.9	305.6

On FLAX dataset. Significant values are in bold.

To comprehensively evaluate the impact of RAHC-Backbone (RAHC), HTD-Efficient Hybrid Encoder (HTD), and P2-Head (P2) on the performance of the model LEHP-DETR proposed in this study, a series of ablation experiments were performed, and the results are summarized in Table 7. The experiments cover seven different model configurations (A3 to G3) and are compared with the RT-DETR-R18 base model. Experimental results show that the A3 configuration significantly reduces the model size, the number of parameters, and FLOPs while slightly improving mAP50 and significantly increasing FPS. The B3 configuration results in an increase in model size and FLOPs, but results in improvements in precision, recall, and mAP50:95 despite a decrease in FPS. The C3 configuration significantly improves precision, recall, mAP50, and MAP50:95, but FPS is significantly reduced. By combining the configurations of different modules, such as D3, E3, and F3, this study finds that different performance trade-offs can be achieved. For example, D3 achieves high FPS while maintaining high precision and recall; E3 achieves significant performance improvement, but FPS decreases; F3 shows an improvement in accuracy and mAP, but still has a low FPS. When all components are introduced together, namely G3 configuration, compared with the base model RT-DETR-R18 the model size is reduced by 66.3%, the number of parameters is reduced by 67.3%, the floating-point calculation is reduced by 37.6%, the precision is increased by 1.0%, the recall rate is increased by 3.2%, and the mAP50 is increased by 2.6%. mAP50:95 is 3.5% higher and, despite the decrease in FPS, it still meets the requirements of real-time detection. These results reveal the contribution of each component to improving the performance of the LEHP-DETR model and demonstrate the performance tradeoff under different component combinations.

**Table 7:** Effect of each module in the LEHP-DETR model on model performance

Model	RAHC	HTD	P2	Model Size (M)	Params (M)	FLOPs (G)	Precision (%)	Recall (%)	mAP50 (%)	mAP50:95 (%)	FPS bs = 16
RT-DETR-R18	×	×	×	38.6	19.9	56.9	95.9	92.0	95.4	57.8	391.6
A3	✓	×	×	22.5	11.3	**34.1**	95.8	92.0	95.6	56.6	**441.4**
B3	×	✓	×	39.2	20.2	61.5	96.1	92.2	95.6	57.9	305.6
C3	×	×	✓	36.4	21.0	78.7	96.5	94.1	97.5	60.6	250.3
D3	✓	✓	×	23.1	11.6	39.7	96.1	92.2	95.6	55.9	343.8
E3	✓	×	✓	23.8	12.0	59.3	**97.0**	95.1	**98.0**	61.1	239.0
F3	×	✓	✓	29.1	15.0	57.9	96.7	94.4	97.5	60.1	238.1
G3	✓	✓	✓	**13.0**	**6.5**	35.5	96.9	**95.2**	**98.0**	**61.3**	260.3

RAHC denotes RAHC-BackBone, HTD denotes HTD-Efficient Hybrid Encoder, and P2 denotes P2-Head. On FLAX dataset. Significant values are in bold.

## Discussion

Flax, as a functional crop rich in essential fatty acids and nutrients, occupies an important position in the field of nutrition and industry. However, the process of flax seed examination is still mainly dependent on manual operation, which is not only inefficient but also error-prone. Advances in computer vision and deep learning techniques, especially in object detection, have provided new possibilities for the automatic detection of flax capsules. In this study, an improved model LEHP-DETR based on RT-DETR was proposed to address the challenge of small object detection in flax capsules. By introducing the RepNCSPELAN4 module, ADown module, Context Aggregation module, and TFE module, as well as the newly designed HWD-ADown module, HiLo-AIFI module, and DSSFF module, this study significantly improved the accuracy and recall. The design of the HWD-ADown module aims to effectively preserve the critical edge information while reducing the image resolution.

The integration of the RepNCSPELAN4 and ADown modules significantly boosts feature representation and diminishes the resolution of the feature map, consequently decreasing computational complexity and augmenting the model’s efficacy in detecting small objects. The introduction of the ContextAggregation module effectively integrates the features from different modules and enhances the ability of the model to utilize the context information of the objects in the image. The HiLo-AIFI module can capture different frequency features in images more comprehensively and optimize the allocation and calculation method of attention heads, which gives the model a better generalization ability for different types of images and scenes. The DSSFF module enhances the model’s capacity for multiscale feature extraction and detailed information retention by fusing feature maps across various scales. Concurrently, the TFE module intensifies the detection capability for dense small objects by concatenating features of large, medium, and small sizes in the spatial dimension, thereby capturing intricate details of flax capsules.P2 detection head aims at the occlusion problem caused by small size and dense distribution in flax capsule object detection task, which can capture the characteristics of small objects more accurately, making it more reliable in practical applications.

In summary, the LEHP-DETR model proposed in this study, through innovative module design and improvement, significantly improves the detection performance of small objects and effectively solves the problem of the flax seed examination. In the experiment of flax dataset, the model size is 13.0M, the number of parameters is 6.5M, FLOPs is 35.5, the precision is 96.9, the recall rate is 95.2, and the average precision mAP50 and MAP50:90 are 98.0% and 61.3%, respectively, which significantly exceeds other comparison models. At the same time, compared with the basic model, the parameter number of the LEHP-DETR is reduced by 67.3%, the model size is reduced by 66.3%, and the FLOPs is reduced by 37.6%. The average detection accuracy of mAP50 and MAP50:95 are increased by 2.6 and 3.5 percentage points, respectively. This research result provides new technical support for the development of the flax industry and also provides new ideas and methods for small object detection tasks in the agricultural field.

### Limitations of the study

Although the improved algorithm in this study achieves excellent object detection performance while remaining lightweight and efficient, this new model LEHP-DETR also has some limitations. On the one hand, the improved algorithm in this study achieved significant performance improvement on the task of detecting small objects in the capsules of flax plants but may have some limitations on other types of object detection tasks. On the other hand, the improved algorithm in this study is relatively inefficient in terms of time efficiency when processing a single image and may not be applicable to scenarios such as edge computing. Therefore, future work in this study is mainly based on the following considerations: (1) Further enhance the computational efficiency and bolster the generalization capabilities of the model to adapt to a wider range of object detection tasks and (2) detect the number of split stems of flax plants as well as branch length and other factors to provide more convenience for flax seed inspection.

## Resource availability

### Lead contact

Requests for further information and resources should be directed to the primary contact, Junying Han (hanjy@gsau.edu.cn).

### Materials availability

This study did not generate new unique reagents.

### Data and code availability

• The FLAX dataset reported in this paper is available from the lead contact upon request.
• The DOTA dataset has been published in a publicly accessible repository. The access address is listed in the key resources table. Datasets are publicly accessible.
• All code associated with this paper can be freely accessed and downloaded via https://github.com/ShawnWang04/LEHP-DETR.
• Any additional information required to reanalyze the data reported in this paper is available from the lead contact upon request.

## Acknowledgments

Thanks to the 10.13039/501100001809National Natural Science Foundation of China (No. 32360437) and the Innovation Fund for Higher Education of Gansu Province (No. 2021A-056), and the National Industrial Technology System of Characteristics Oil of China, MOF and MARA (CARS-14-1-05) for supporting this project.

## Author contributions

C.W. designed and implemented experiments and wrote the paper; J.H. reviewed and guided the paper; C.L. provided funding acquisition; J.Z. and Y.Q. provided flax images and Funding acquisition.

## Declaration of interests

The authors declare no competing interests.

## STAR★Methods

### Key resources table

**Table:** 

REAGENT or RESOURCE	SOURCE	IDENTIFIER
**Deposited data**		

Flax Datasets	This paper	N/A
DOTA Datasets	Xia et al.^32^	https://captain-whu.github.io/DOTA/index.html

**Software and algorithms**		

YOLOv5	Ultralytics company	https://github.com/ultralytics/ultralytics/tree/main/ultralytics/cfg/models/v5
YOLOv8	Ultralytics company	https://github.com/ultralytics/ultralytics/tree/main/ultralytics/cfg/models/v8
YOLOv9	Wang et al.^20^	https://github.com/ultralytics/ultralytics/tree/main/ultralytics/cfg/models/v9
RT-DETR	Zhao et al.^25^	https://github.com/lyuwenyu/RT-DETR/tree/main/rtdetr_pytorch
LEHP-DETR	This paper	https://github.com/ShawnWang04/LEHP-DETR
PyTorch	Version 2.2.1	https://pytorch.org/docs/2.2/
Python	Version 3.8.18	https://www.python.org/downloads/release/python-3818/
CUDA	Version 12.4	https://docs.nvidia.com/cuda/cuda-toolkit-release-notes/index.html

### Experimental model and study participant details

#### Experimental platforms

The experimental setup for this study includes a Windows 11 64-bit Professional OS, an Intel Core i7-13700 13th Gen CPU, an NVIDIA GeForce RTX 4090 GPU with 24 GB VRAM, 64 GB DDR4 RAM at 4800 MHz, Python 3.8.18 for programming, Pytorch 2.2.1 as the deep learning framework, and CUDA 12.4 for GPU acceleration.

#### Model training parameters

The model training employs Pytorch’s Ultralytics version, with input images resized to 640 × 640. Training parameters include 8 workers, a batch size of 16, 380 epochs, the AdamW^31^ optimizer with a momentum of 0.9 and weight decay of 0.0001, and an initial learning rate of 0.0001, without utilizing pre-trained weights.

#### Flax datasets

In this study, a flax plant dataset containing 500 flax varieties was created, covering data from two consecutive years in 2021 and 2022. The plants of the same crop in different planting areas will undergo a certain degree of decoration. In order to ensure the accuracy and generalization of the model, each variety selects one plant in each of the three different planting areas and takes one image each. Each variety has a total of three images. These images were collected from four flax experimental stations in China: Zhangye Experimental Site in Gansu (100.37E, 38.84N), Jingtai Experimental Site in Gansu (104.07E, 37.18N), Lanzhou New District Experimental Site in Gansu (103.70E, 36.56N) and Gansu Academy of Agricultural Sciences Inner Experimental Site (103.68E, 36.09N), and these latitudes and longitudes were determined based on the WGS84 coordinate system. This study used a Microvision Manufacturing Technology Corporation (Microvision) MV-HS2000GM/C2 industrial camera for image acquisition, which has a maximum resolution of 5472 × 3648, an optical size of 1 inch, a pixel size of 2.4 × 2.4, a CMOS sensor type, an exposure time of 40 μs to 2 s, and a data bit depth of 8/12 of the data bit depth, and a maximum frame rate of 5 fps. Images of flax plants with a resolution of 5472 × 3000 pixels were taken, and a total of 2157 images were acquired. The acquisition process is shown in Figure 9. In this study, we used the LabelImg tool to annotate capsules in flax images and stored the annotation results in Pascal VOC dataset format. There are 2157 original images of flax plants, which are divided into training set, validation set and test set according to the ratio of 7:1:2. In order to enhance the scale and diversity of the dataset, we only implemented data augmentation on 1509 flax plant images in the training set. As shown in Figure 10, the training dataset was able to be expanded to 3700 images through the application of random and mixup^30^ augmentation techniques.

**Figure:**
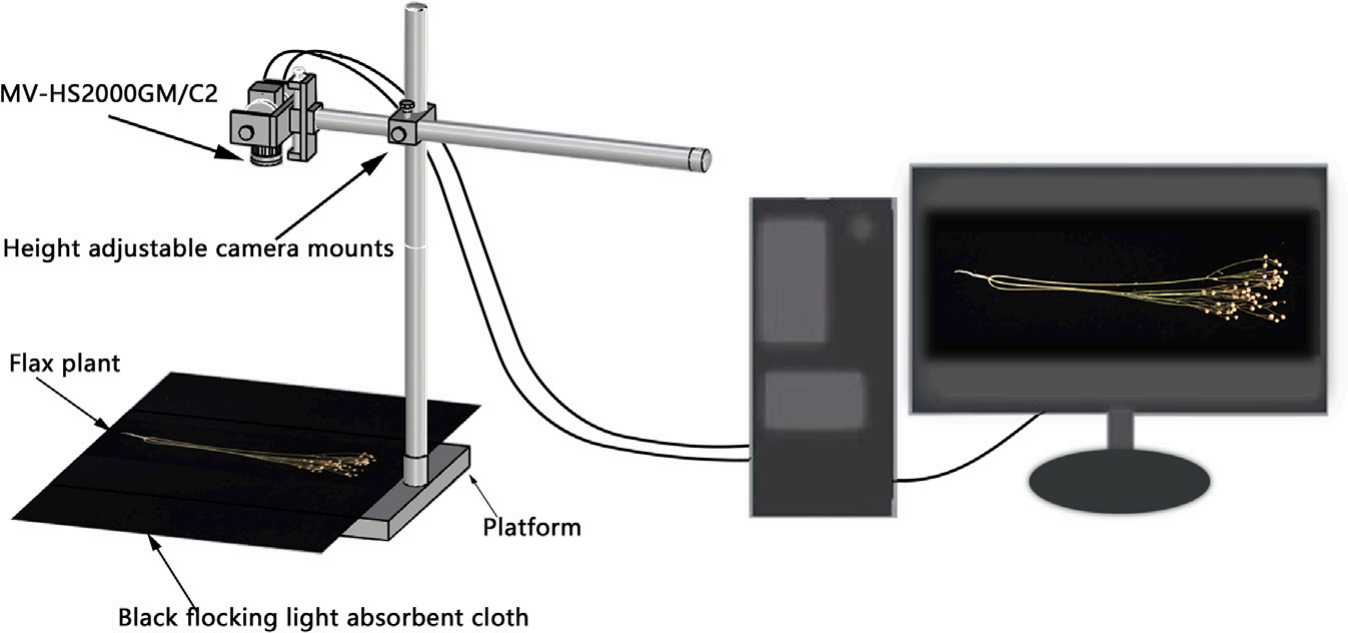
Flax data collection process

**Figure:**
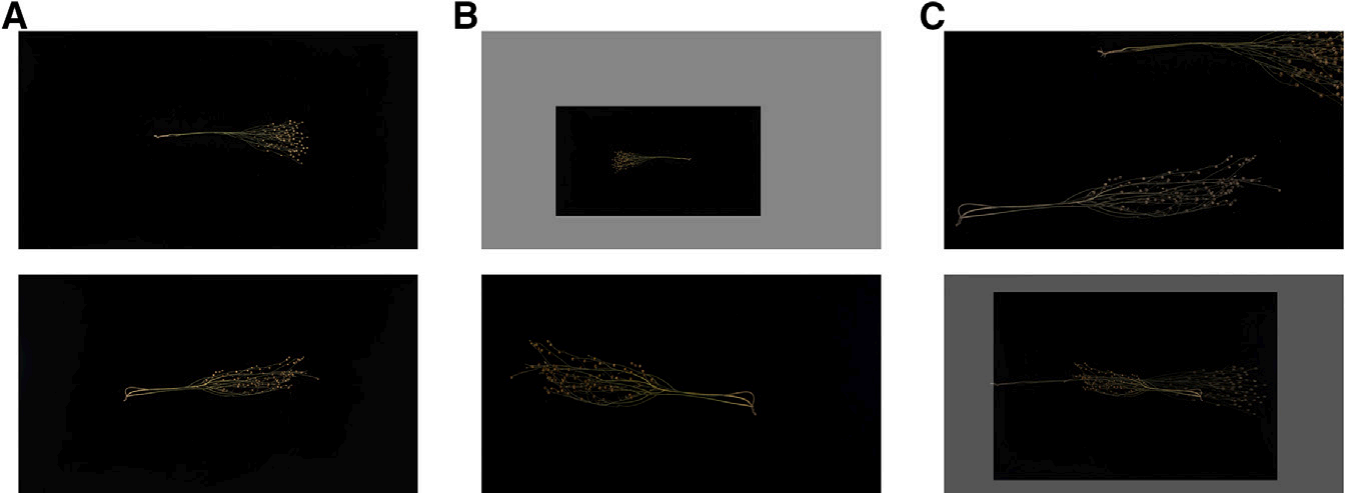
Data augmentation example (A) original image (B) random augmentation image (C) mixup augmentation image.

#### DOTA datasets

DOTA^32^ is a large-scale dataset for object detection in aerial images. The images are collected from different sensors and platforms. Each image is of the size in the range from 800 × 800 to 20,000 × 20,000 pixels and contains objects exhibiting a wide variety of scales, orientations, and shapes. DOTA-v1.0 contains 15 common categories, 2806 images and 188282 instances. In order to train efficiently, the dataset is divided into 2048 × 2048 with a step size of 500.The training set and validation set in the split data are 6214 and 2163 respectively.

#### Assessment of indicators

This study uses Model Size, Parameters, FLOPs, Precision, Recall, mAP, and FPS as evaluation metrics.

Model Size: indicates the size of the model file, which reflects the complexity of the model and the amount of memory required; the smaller, the better. The unit is M.

Parameters: indicates the learnable variables in the model, such as weights and biases. The number of parameters measures model complexity and computational cost; the lower, the better. Unit is M.

FLOPs: Denotes the floating point operations (FLOPs) needed for the model to process an input. The lower, the better. Unit G.

Precision: Reflects the model’s accuracy in predicting positive samples, or the likelihood that a predicted bounding box encloses a true object. Superior performance is indicated by a higher value, with TP denoting True Positive and FP denoting False Positive. As shown in Equation 1.

(Equation 1) $$Precision=\frac{TP}{TP+FP}$$

Recall: Represents the ratio of correctly predicted positive samples to the total true positives, denoting the model’s ability to detect real objects. A higher value signifies better performance, with FN denoting False Negative. As shown in Equation 2.

(Equation 2) $$Recall=\frac{TP}{TP+FN}$$

mAP^33^: Represents the model’s mean accuracy across various thresholds, providing a holistic performance metric, with higher values indicating superior performance. $AP_{i}$ denotes the average precision at the i-th threshold, and N is the number of categories and AP is the average precision of each category. The formula is given in 3.

(Equation 3) $$mAP=\frac{1}{N}\sum_{i=1}^{N}AP_{i}$$

mAP50: Refers to the average precision (mAP) at an Intersection over Union(IoU) threshold of 0.5.

mAP50:95: A more comprehensive evaluation metric that calculates and averages the mAPs at a total of 10 IoU thresholds from IoU = 0.5 to IoU = 0.95 in steps of 0.05. This evaluation method can more comprehensively reflect the detection ability of the model under different difficulties.

FPS: The model’s frame processing time, or t, reflects its real-time performance, with lower values indicating higher efficiency. The formula is given in 4.

(Equation 4) $$FPS=\frac{1}{t}$$

### Method details

#### RAHC-BackBone structure

In the BackBone structure, this study takes an innovative approach by replacing the traditional ResNet^34^ structure with the RepNCSPELAN4 module, the ADown module, and the HWD-ADown module. The purpose of this modification is to optimize the computational complexity and number of parameters of the model, thus making it lighter and more efficient. Details of this architecture are shown in Figure 1. While the ResNet architecture enhances the depth and expressiveness of the model through residual connectivity, its high number of parameters and computational complexity, as well as single-scale feature extraction, limit its application in small object detection. To overcome these limitations, this study employs the RepNCSPELAN4 module, which integrates the RepNCSP module used for feature extraction and the ELAN^35^ module used for enhanced object detection, thereby improving the accuracy of detection. The ADown module is mainly used for feature extraction and downsampling operations, where the ADown module is able to preserve the details and important features that are crucial for feature fusion and feature transfer. The design of the HWD-ADown module combines the ADown module and the Haar Wavelet downsampling^36^ module. As shown in Figure 11, the module is designed with significant advantages in preserving image details and edge information, which is crucial for object localization and recognition. In the HWD-ADown module, the Haar wavelet downsampling module is used in this study to replace the 3x3 convolution in the ADown module. In order to reduce the computational complexity and the number of parameters of the model, only the last ADown module in BackBone is replaced by the HWD-ADown module in this study.

**Figure:**
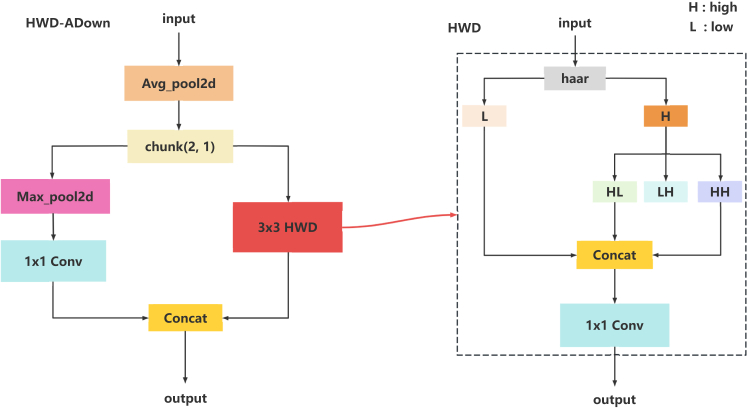
HWD-ADown structure

#### HTD-Efficient Hybrid Encoder structure

In the HTD-Efficient Hybrid Encoder structure, this study designs the HiLo-AIFI module and DSSFF module and introduces the TFE module. As shown in Figure 12, in the HiLo-AIFI module, this study innovatively replaces the traditional Multi-head Attention module in AIFI with the HiLo Attention^37^ module, which mainly relies on the self-attention mechanism, and for the small object detection task, the small object usually occupies only a small area in the image and therefore contains valid information. A very small area in the image and, therefore, contains very limited effective information. This makes it difficult for Multi-Head Attention to effectively capture the features of small objects. In contrast, HiLo Attention divides attention into two parts: Low Frequency and High Frequency. The Low-Frequency part focuses on capturing global structural information, while the High-Frequency part focuses on extracting local detail information. This mechanism of separating and processing different frequency information enables HiLo Attention to capture and exploit contextual information in the image more effectively, especially in small object detection tasks, where it can more accurately handle the relationship between small objects and complex backgrounds. At the same time, the module is able to capture different frequency features in images more comprehensively, giving the model a better ability to generalize to different types of images and scenes.

**Figure:**
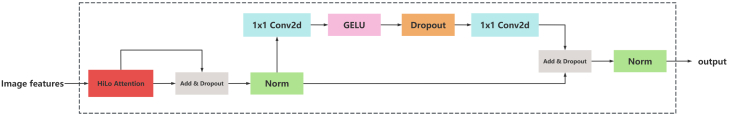
HiLo-AIFI structure diagram

In order to meet the processing needs of features at different scales, to capture and fuse object information at different scales more comprehensively, to obtain richer contextual information, and to improve the accuracy and robustness of detection, the DSSFF module is designed in this study, as shown in Figure 13. This module combines the Scale Sequence Feature Fusion Module(SSFF)^28^ with the DySample^38^ module and replaces the Upsample module in the SSFF module with the DySample module. The traditional Upsample module achieves the increase in feature map size by interpolation or copy operations, which is simple and fast to compute but has limitations in terms of detail retention and edge preservation. In contrast, the DySample module is able to better preserve the edge detail information of the image, and is able to detect and locate small objects more accurately. In addition, DySample facilitates the adaptive modification of the feature map dimensions in response to the specific requirements of diverse tasks, thus improving the performance of the model. The output features extracted from the RAHC-backbone by the DSSFF module are fused by the HTD-Efficient Hybrid Encoder section shown in Figure 1. By effectively fusing these feature maps, flax capsules with different spatial scales, sizes, and shapes can be captured. In DSSFF, different feature maps are normalized to the same size and superimposed by dynamic Upsampling as input to 3D convolution to combine multi-scale features.

**Figure:**
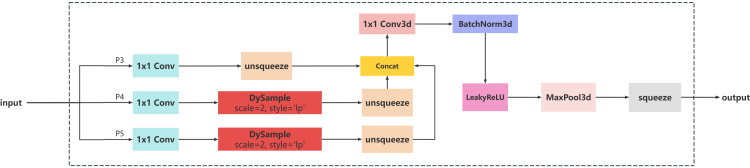
DSSF module structure

The TFE module is also introduced in this study. The structure is shown in Figure 14. Due to the varying dimensions across the feature layers of the RAHC-backbone architecture, the traditional feature pyramid network (FPN) fusion mechanism only up-samples the feature maps with small dimensions and adds them to the previous feature layer, which may result in overlooking the rich detail information in the feature layers with larger dimensions. To encapsulate the intricate details of flax capsules and enhance the detection proficiency for densely packed small objects, the TFE module integrates feature maps of three distinct scales Large (L), Medium (M), and Small (S) in the spatial domain. The basic principle of the TFE module acquires the three tensors, L, M, and S, and Utilizing the dimensions of the M tensor for the determination of object sizes and adjusts the L by adaptive pooling operation to make the L the same size as M by adaptive pooling operation, and perform pooling and then sum up. Use the interpolation operation to adjust S to the same size as M. Pool L, M, and S into a tensor according to the channel dimension (dim = 1). The formula is shown in (Equation 5). Where FTFE is the feature map output from the TFE module, Fl, Fm, and Fs are the feature maps of large, medium, and small dimensions respectively. FTFE is obtained by splicing Fl, Fm, and Fs, FTFE has the same resolution as Fm, and the number of channels is three times that of Fm.

(Equation 5) $$F_{TFE}=Concat\left(F_{l},F_{m},F_{s}\right)$$

**Figure:**
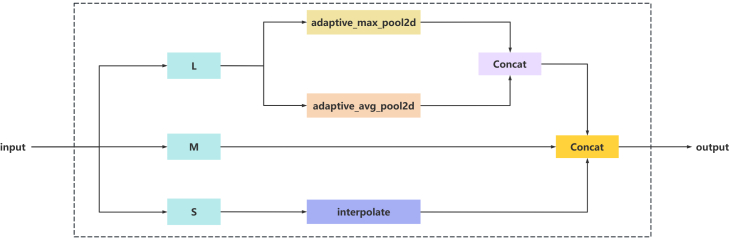
TFE module structure diagram L for Large, M for Medium, S for Small.

#### P2-head structure

To address the challenges of flax object detection, this study focuses on the problem of occlusion due to the small size and dense distribution of the objects. The feature information of such objects is often lost after multiple downsampling, making it difficult to detect them effectively even when using a P3-layer detection head with higher resolution. To overcome this difficulty, this study introduces a new detection head based on P2 layer features in the head part, whose structure is shown as the P2 head in Figure 1. The P2 layer detection head has a resolution of 160 × 160 pixels, which means that only two downsampling operations are performed in the backbone network, and thus richer underlying feature information can be preserved. Through the incorporation of the P2 detection head into the model architecture, this investigation achieves a substantial enhancement in the detection accuracy for small objects. The P2 detection head is designed to extract features of small objects with greater precision, thereby enhancing the model’s generalization capacity and ensuring its robustness in real-world scenarios.
